# SNORD47, a box C/D snoRNA, suppresses tumorigenesis in glioblastoma

**DOI:** 10.18632/oncotarget.16693

**Published:** 2017-03-30

**Authors:** Bin Xu, Min-Hua Ye, Shi-Gang Lv, Qi-Xue Wang, Miao-Jing Wu, Bing Xiao, Chun-Sheng Kang, Xin-Gen Zhu

**Affiliations:** ^1^ Department of Neurosurgery, The Second Affiliated Hospital of Nanchang University, Nanchang, Jiangxi, China; ^2^ Department of Neurosurgery, Tianjin Medical University General Hospital, Heping District, Tianjin, China; ^3^ Laboratory of Neuro-Oncology, Tianjin Neurological Institute, Tianjin, China

**Keywords:** SNORD47, glioblastoma, cell cycle, proliferation, invasion

## Abstract

SNORD47 is a member of the C/D box small nucleolar RNAs, which have been implicated in cancer development. We intended to investigate the therapeutic potential of SNORD47 in glioma. We found that the expression of SNORD47 was downregulated in glioma tissues samples and inversely associated with advanced tumor stage (WHO grade IV). Kaplan-Meier survival analysis revealed that glioma patients with high SNORD47 expression had longer overall survival than those with low SNORD47 expression. SNORD47 suppressed the proliferation of glioma cells and induced G2 phase arrest. In addition, upregulation of SNORD47 suppressed invasion and epithelial-mesenchymal transition in glioma cells, and combination treatment with lenti-SNORD47 could augment the anti-tumor effect of temozolomide. These results showed that SNORD47 acted as a tumor suppressor in glioma, and provided the potential anti-tumor function in glioma treatment.

## INTRODUCTION

Gliomas are the most prevalent primary tumors in the central nervous system. Glioblastoma, World Health Organization (WHO) grade IV, is the most aggressive type of brain tumor. Patients afflicted with glioblastoma have a median survival period of 12 to 15 months [1]. Although there are some treatment options, including maximum surgical resection, postoperative radiotherapy and chemotherapy, the prognosis of glioblastoma patients remains very poor. Temozolomide, as a first-line chemotherapy drug for treating glioblastoma, was introduced to improve the outcome to some extent. However, the drug resistance limited the effectiveness of temozolomide treatment in glioblastoma. Thus, there is an urgent need to identify the underlying molecular pathological mechanism of glioma and develop new therapeutic targets to improve the quality of life and prolong the survival time of glioblastoma patients.

Small nucleolar RNAs (snoRNAs), as a subset of non-coding RNAs, is approximately 60–300 nucleotides in length. SnoRNAs have been shown to guide the modification of other RNAs, primarily rRNA, tRNA and snRNA. There are two main classes of snoRNA, the C/D box snoRNAs, which are associated with methylation, and the H/ACA box snoRNAs, which are associated with pseudouridylation. There is compelling evidence suggesting that snoRNAs are not only involved in the physiological functions of normal cells but also in underlying mechanisms regulating the progression and development of cancer. For example, snoRNA U50, which is frequently deleted in prostate cancer and breast cancer, could suppress the cancer progression [2, 3]. Other studies also have suggested that snoRNAs can be characterized as oncogenic or suppressive molecular RNAs in the cancer progress [4–9]. However, there have been few studies focusing on the function of snoRNA in glioma.

Hox Transcript Antisense Intergenic RNA (HOTAIR), a potential biomarker of glioblastoma, is highly expressed in glioblastoma and is associated with poor prognosis [10]. In our previous study, we discovered that downregulating HOTAIR resulted in numerous transcriptome alterations, including the upregulation of C/D box SNORD47. SNORD47 is a small nuclear RNA excised from the tenth intron of growth arrest-specific transcript 5 (GAS5) pre-RNA (data not shown). Because of this inverse correlation between HOTAIR and SNORD47 expression, we postulated that SNORD47 may be characterized as a suppressive molecule in glioblastoma. Hence, we performed a series of assays to test this hypothesis. In this study, we observed lower SNORD47 expression in clinical glioblastoma specimens and longer survival time in patients with higher SNORD47 expression. Our results showed that SNORD47 significantly inhibited the proliferation, colony formation, migration and invasion abilities of glioblastoma cells and induced a G2-phase arrest. Furthermore, we found a potential synergistic effect between SNORD47 and temozolomide *in vivo* and *in vitro*. These results showed that SNORD47 acted as a tumor suppressor in glioblasoma.

## RESULTS

### The expression of SNORD47 is downregulated in glioblastoma and is inversely correlated with the glioma progression

**Figure 1:**
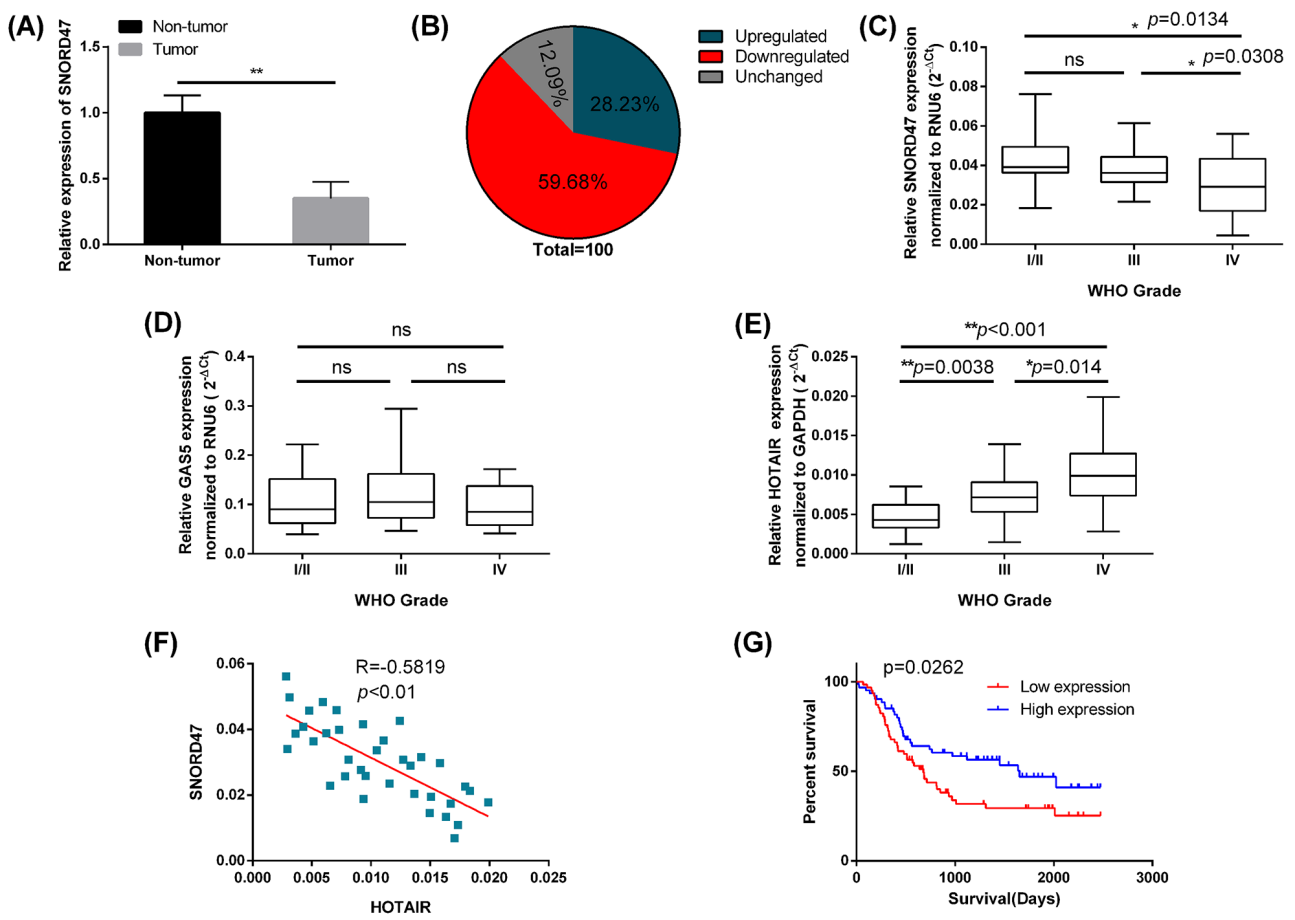
The low SNORD47 expression was associated with poor prognosis in glioma patients **(A)** Relative expression level of SNORD47 in glioma tissues and adjacent non-tumor tissues was measured by qRT-PCR (N=124). **P <0.01 (paired t-test). **(B)** The percentage of glioma patients with SNORD47 expression level (upregulated, downregualted and unchanged). **(C, D, E)** SNORD47, GAS5 and HOTAIR expression levels in different glioma clinical grades were detected by qRT-PCR. *P<0.05, **P <0.01 (ANOVA); ns, no significant difference (*P*≥0.05) relative to the other group. **(F)** The correlation between HOTAIR and SNORD47 in Grade IV glioma specimens. R=-0.5819, **P<0.01 (Pearson correlation analysis). **(G)** The survival of glioma patients with higher or lower expression of SNORD47 were demonstrated by Kaplan-Meier survival curves. *P*=0.0262 (Log-rank (Mantel-Cox) test).

To investigate the potential role of SNORD47 in glioma, we used qRT-PCR to measure the expression of SNORD47 in 124 clinical glioma specimens and adjacent normal tissues. The results showed that the expression of SNORD47 was significantly lower in glioma specimens compared to the adjacent normal tissues (Figure 1A). Exactly 59.68% (74 out of 124) glioma tissues showed downregulated expression of SNORD47 compared with adjacent normal tissues, while 12.09% (15 out of 124) glioma tissues showed no change (Figure 1B). Furthermore, we examined the expression of SNORD47, GAS5, HOTAIR in 124 clinical glioma specimens of different grades: WHO grade I/II (n=40), grade III (n=48), and grade IV (n=36). The results showed that the expression of SNORD47 was significantly decreased in grade IV glioblastoma compared with the lower-grade glioma specimens (Figure 1C). However, there was no significant difference in the expression of SNORD47 between the grade I/II and grade III glioma specimens (Figure 1C). There were no significant differences in the expression of GAS5 among the different grades of glioma specimens (Figure 1D). The expression level of HOTAIR was positively associated the grades of the glioma specimens (Figure 1E). The differences among the different grades were significant (Figure 1E). And the correlation between HOTAIR and SNORD47 was significantly inverse (Figure 1F).

Next, we study the relationship between the expression of SNORD47 and clinicopathological parameters in glioma patients. The results showed that the lower expression of SNORD47 was significantly associated with the advanced WHO grade (III/IV) (Table 1). Furthermore, Kaplan-Meier survival analysis indicated that patients with higher SNORD47 expression in glioma tissues had better survival than those with lower SNORD47 expression (Figure 1G). Taken together, these results revealed that SNORD47 was aberrantly down-regulated in glioma tissues, and this down-regulation was associated with glioma progression.

**Table 1:** Association between SNORD47 expression and clinicopathological features in glioma

Patients(n=124)	N of cases	Relative SNORD47 expression		*P* value
		Low	High	
**Gender**				
Male	80	52(65.0%)	28(35.0%)	0.879
Female	44	28(63.6%)	16(36.4%)	
**Age(years)**				
<55	56	36(64.3%)	20(35.7%)	0.534
≥55	68	40(58.8%)	28(41.2%)	
**Tumor size(cm)**				
<5	86	48(55.8%)	38(44.2%)	0.955
≥5	38	21(55.3%)	17(44.7%)	
**Tumor Grade**				
I/II	54	26(48.1%)	28(51.9%)	**0.008**
III/IV	70	50(71.4%)	20(28.6%)	

### Overexpression of SNORD47 inhibited cell proliferation and colony formation

**Figure 2:**
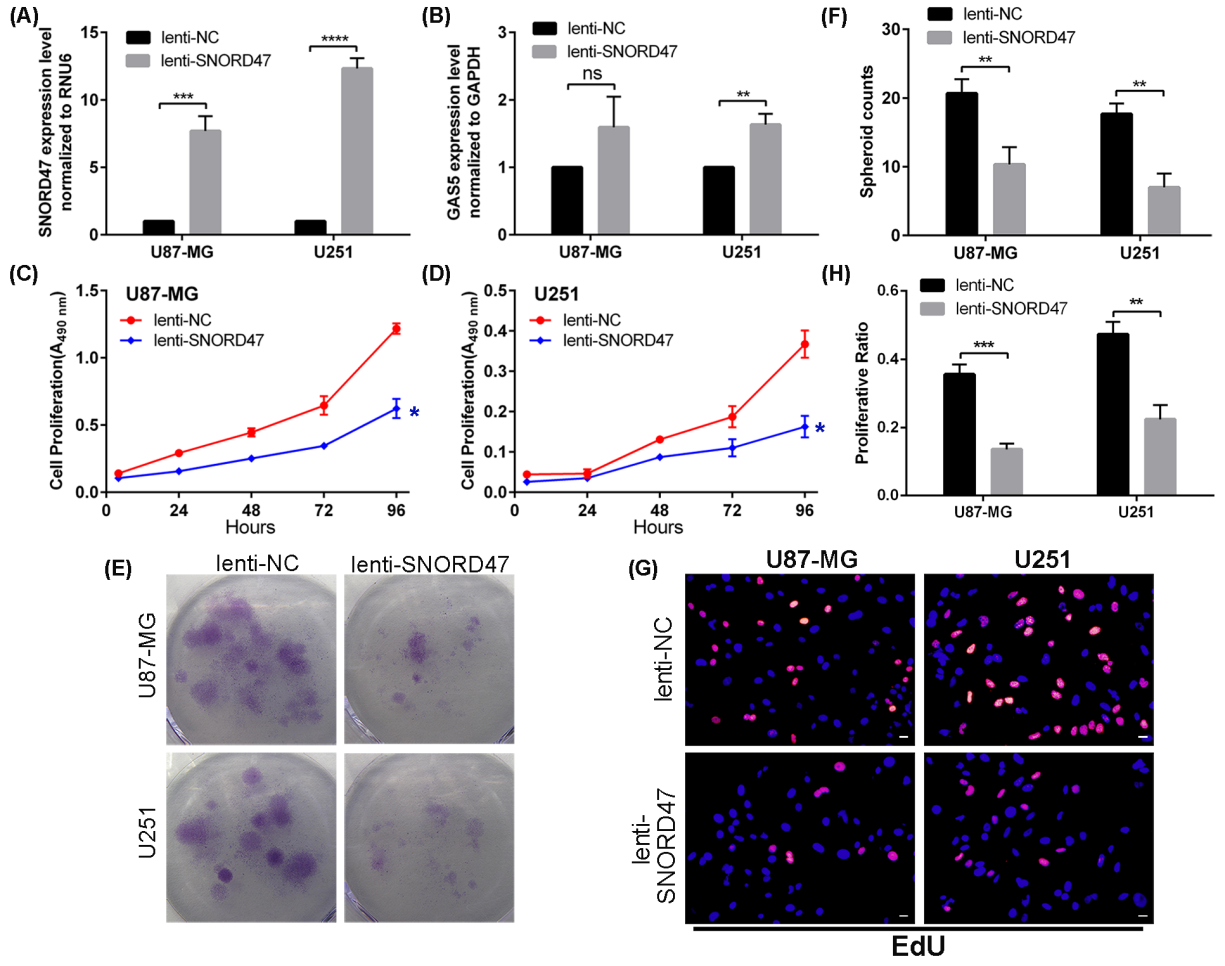
SNORD47 suppressed the proliferation in glioma cell lines SNORD47 **(A)** and GAS5 **(B)** were measured by qRT-PCR after transfection with lenti-NC or lenti-SNORD47 in U87-MG and U251 glioma cell lines. The CCK-8 assay was employed to test the proliferative ability of U87-MG **(C)** and U251 **(D)** cell line with or without SNORD47 overexpression. **(E-F)** Clone formation assay was performed to test the cell growth of SNORD47 overexpression cell lines. **(G-H)** EdU assay was performed to study the proliferation of glioma cells. Representative histogram exhibited the total spheroid numbers in 3 randomly chosen fields in the control and treated group. *P < 0.05, **P<0.01, ***P<0.001 (Student's t test) compared with the control group. Bar =20μm.

As previously described, SNORD47 may exhibit anti-cancer effects. Therefore, we performed a series of assays to determine whether SNORD47 had an impact on the biological functions of glioblastoma. First, we transfected U87-MG and U251 cells with lenti-SNORD47. After a 48 h transfection, qRT-PCR results showed that the expression of SNORD47 was substantially increased by approximately 8-fold and 12-fold in U87-MG and U251 cells, respectively (Figure 2A). Unexpectedly, the GAS5 expression level in both cell lines was also elevated by approximately 1.6-fold (Figure 2B). Furthermore, we performed the following experiments to investigate the biological role of SNORD47 in glioma. A CCK-8 assay demonstrated that the proliferative capacity of the treated group was clearly gradually inhibited compared with the control group in the U87-MG and U251 glioma cell lines (Figure 2C, 2D). In addition, a colony formation assay also indicated that overexpression of SNORD47 inhibited proliferation in U87-MG and U251 cells compared with the control group (Figure 2E, 2F). This result was consistent with the CCK-8 assay. What's more, after treated with lenti-SNORD47, the EdU assay indicated that the proliferation of glioma cells was significantly inhibited (Figure 2G–2H). These results indicated that overexpression of SNORD47 was capable of suppressing the proliferation and growth abilities of U87-MG and U251 cells.

### Overexpression of SNORD47 induced a G2 phase arrest and impaired the invasive capacity

**Figure 3:**
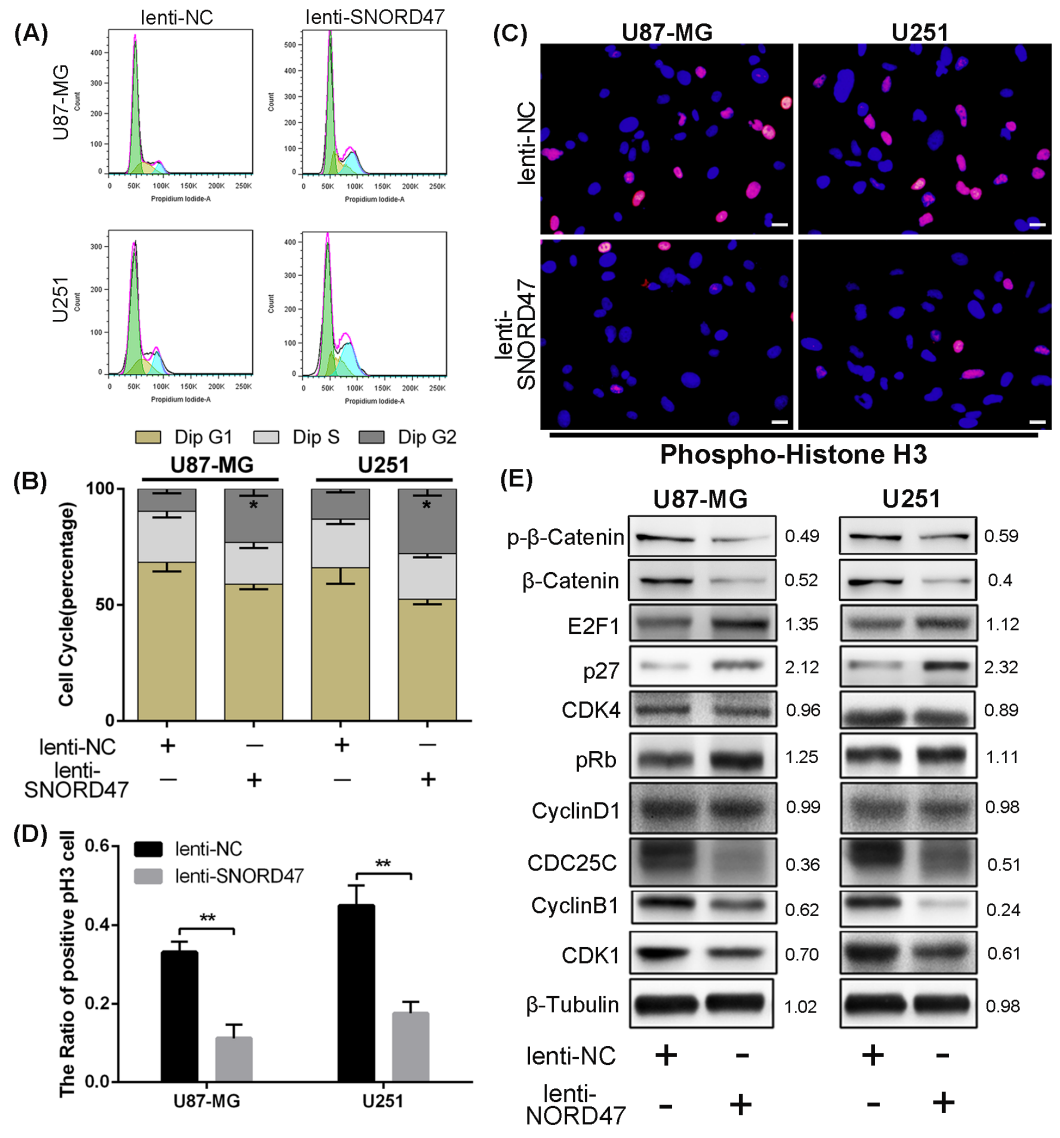
SNORD47 induced a G2-phase arrest in U87-MG and U251 cell line **(A, B)** The cell cycle of NC and SNORD47 overexpression cells was analysed by flow cytometry. **(C-D)** phosphorylated-H3 was examined by immunofluorescence in lenti-NC and lenti-SNORD47 treated glioma cells. **(E)** Cell cycle check point proteins were measured by western blot in lenti-NC and lenti-SNORD47 overexpression cells. *P < 0.05, **P<0.01 (Student's t test) compared with the control group. Bar =20μm.

**Figure 4:**
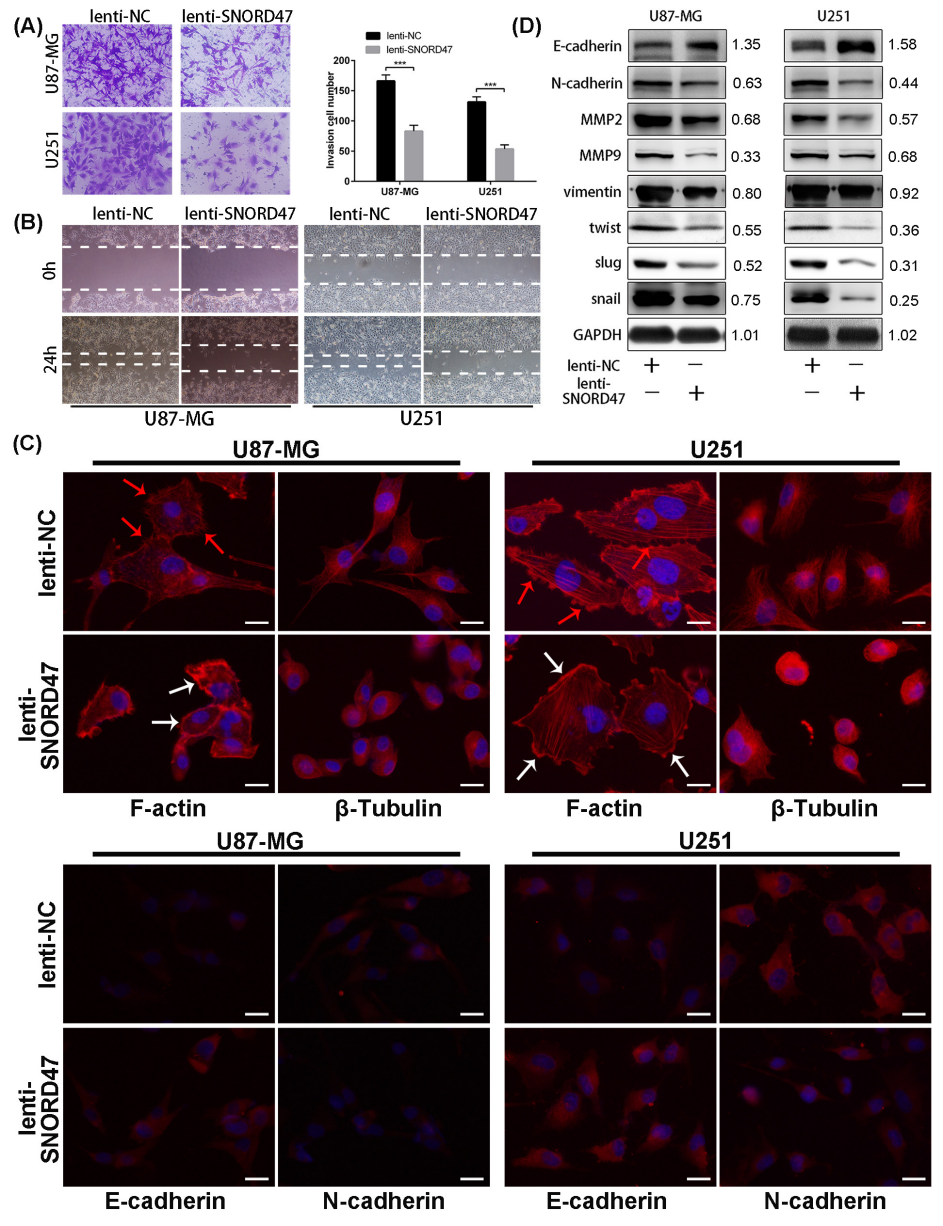
SNORD47 suppressed the invasion and epithelial-to-mesenchymal in glioma cells **(A)** Invasion ability of glioma cells with or without SNORD47 overexpression glioma cells was measured by transwell assay. ***P<0.001 (Student's t test). **(B)** The cell migration of U87-MG and U251 cell line was examined by wound healing assay. **(C)** The results of F-actin staining displayed a cortical pattern in lenti-SNORD47-treated cells and a stress-fiber pattern in Lenti-NC-treated cells. β-Tubulin staining showed a skeleton retraction after treated with SNORD47. Immunofluorescence staining also displayed a lower N-cadherin expression level and a higher E-cadherin expression level after the treatment with lenti-SNORD47. **(D)** Western blot analysis exhibited the expression of EMT-associated proteins in lenti-NC or lenti-SNORD47 treated glioma cells. The red arrow and the white arrow indicatedinvadopodia and lamellipodia respectively. Bar =20μm.

As indicated above, enhanced expression of SNORD47 inhibited the proliferation and growth of glioma cell lines. To explore the possible mechanism of these effects, we evaluated the cell cycle distribution change after treatment with the SNORD47 overexpression lentivirus. The percentage of cells in G2/M phase in the treatment group was markedly increased by approximately 2.3-fold and 2.1-fold in U87-MG and U251 cells, respectively (Figure 3A, 3B). To identify the mitotic cells, we performed immunofluorescence staining with an antibody against phosphorylated Histone H3, which is a marker of mitosis. The result demonstrated a lower proportion of pH3-positive cells in SNORD47-treated cells, which suggested a lower percentage of cells in M phase (Figure 3C–3D). These results revealed a G2 phase arrest as a result of overexpression of SNORD47. Western blot results showed lower expression of G2 phase-related proteins, including CDC25C, CDK1, and CyclinB1, after the treatment, as well as the expression of β-Catenin and p-β-Catenin (Figure 3E). After the overexpression of SNORD47, the expression of E2F1, p27, pRb displayed a slightly increase, while the expression of CDK4, CyclinD1 remained unchanged (Figure 3E). A Transwell assay indicated that the number of cells that crossed the membrane dramatically decreased by approximately 50% after SNORD47 overexpression (Figure 4A). Moreover, in a wound healing assay, after treatment with lenti-SNORD47, fewer U87-MG and U251 cells had migrated into the scratched area after 24 h compared to the control cells, which revealed that the migration ability was significantly suppressed after treatment (Figure 4B). Immunofluorescence staining with F-actin showed an increased number of lamellipodia in lenti-SNORD47-treated cells, while the control group of cells exhibited an elevated number of invadopodia (Figure 4C). The F-actin staining suggested a shift from a stress-fiber pattern to a cortical pattern as a result of enhanced expression of SNORD47 in both U87-MG and U251 cells. β-Tubulin staining of SNORD47-treated cells showed that the cytoskeleton exhibits a retraction from the periphery of the cell to the core of the cell (Figure 4C). Moreover, immunofluorescence staining with antibodies against E-cadherin and N-cadherin, which are Epithelial-Mesenchymal Transition (EMT)-related markers, exhibited an increased expression of E-cadherin and a lower expression of N-cadherin in SNORD47-treated glioma cells (Figure 4C). The western blot analysis was consistent with immunofluorescence staining, showing a significant downregulation of N-cadherin, snail, slug, Vimentin, twist, MMP2, and MMP9 and a higher expression level of E-cadherin after the treatment (Figure 4D). These results strongly indicated that SNORD47 induced a G2-phase arrest and suppressed the invasion in glioma cell lines.

### SNORD47 sensitizes glioblastoma cells to temozolomide

**Figure 5:**
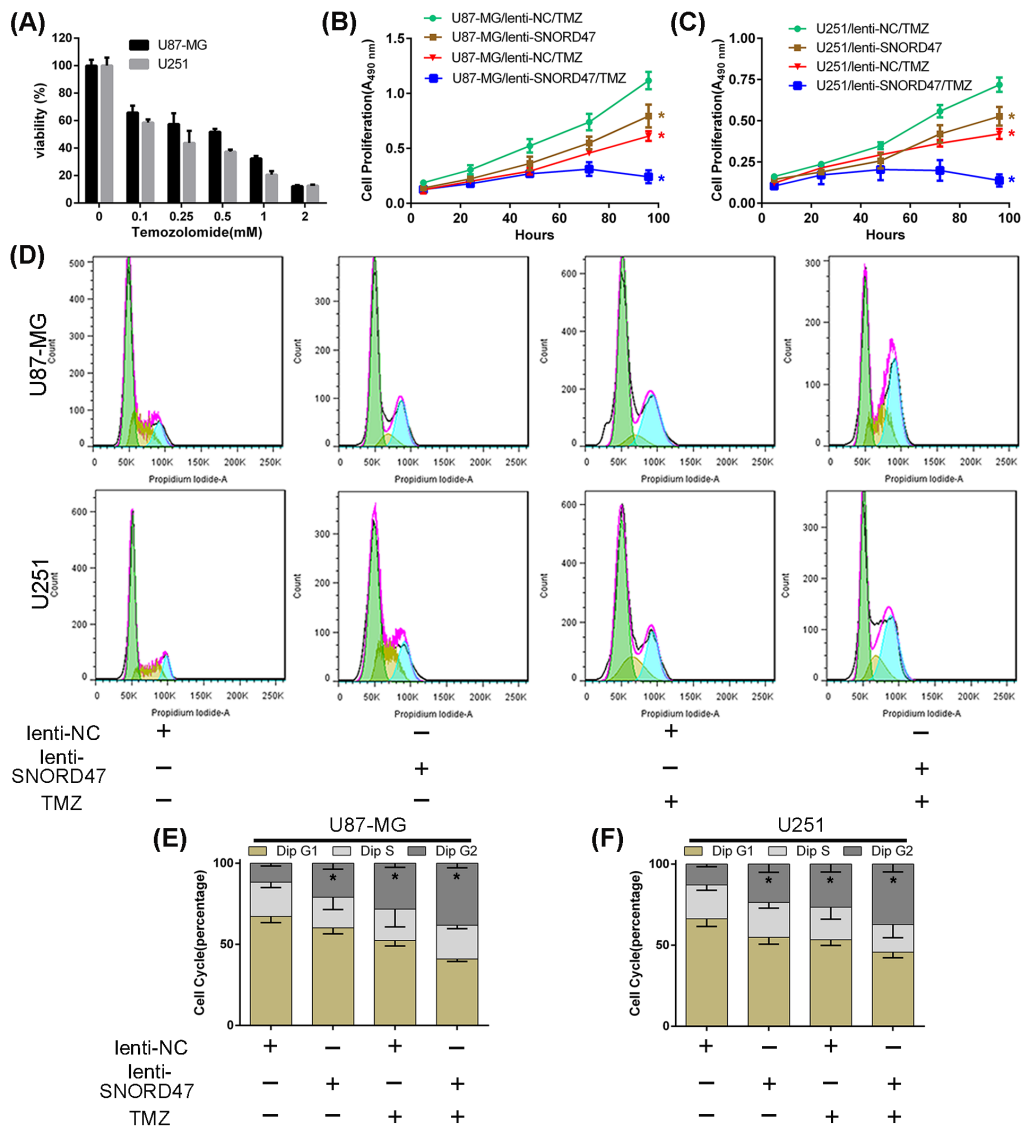
SNORD47 sensitized U87-MG and U251 cells to temozolomide treatment **(A)** The IC50 assay was taken to measure the inhibitory concentration of temozolomide on U87-MG and U251 glioma cells. **(B,C)** The proliferation of NC and SNORD47 glioma cells under temozolomide (200uM) treatment was measured by CCK8 analysis. **(D,E,F)** The cell cycle distribution of NC and SNORD47 glioma cells under temozolomide treatment was examined by flow cytometry. *P < 0.05 (Student's t test) compared with the control group.

To investigate whether SNORD47 enhanced the sensitivity of glioma cells to temozolomide, we used a CCK-8 assay and examined the cell cycle distribution. First, after cells were treated with increasing concentrations of temozolomide for 72 h, the viability in U87-MG and U251 cells was gradually reduced (Figure 5A). After co-treatment with lenti-SNORD47 and temozolomide, the proliferation ability, as measured by CCK-8 assay, was markedly inhibited compared with other groups, including both the SNORD47 overexpression-treated and temozolomide-treated groups, but especially compared to the control group (Figure 5B, 5C). Cells treated with either the SNORD47 overexpression lentivirus or temozolomide alone also exhibited an attenuated capacity of proliferation in comparison with the control group cells. Flow cytometry results showed that after treatment with SNORD47, temozolomide and SNORD47-temozolomide, the proportion of cells in G2 phase was dramatically increased to 21%, 28%, and 38% from 12% in U87-MG cells and significantly augmented to 24%, 27%, and 37% from 13% in U251 cells, respectively, in comparison with control cells (Figure 5D, 5E, 5F). These results strongly indicated that SNORD47 can enhance the therapeutic effects of temozolomide in glioma cells, which suggests that the combination of SNORD47 and temozolomide results in a better inhibition of glioma proliferation and growth.

### SNORD47 inhibited tumorigenesis and enhanced the temozolomide antitumor efficacy in an orthotopic tumor model

**Figure 6:**
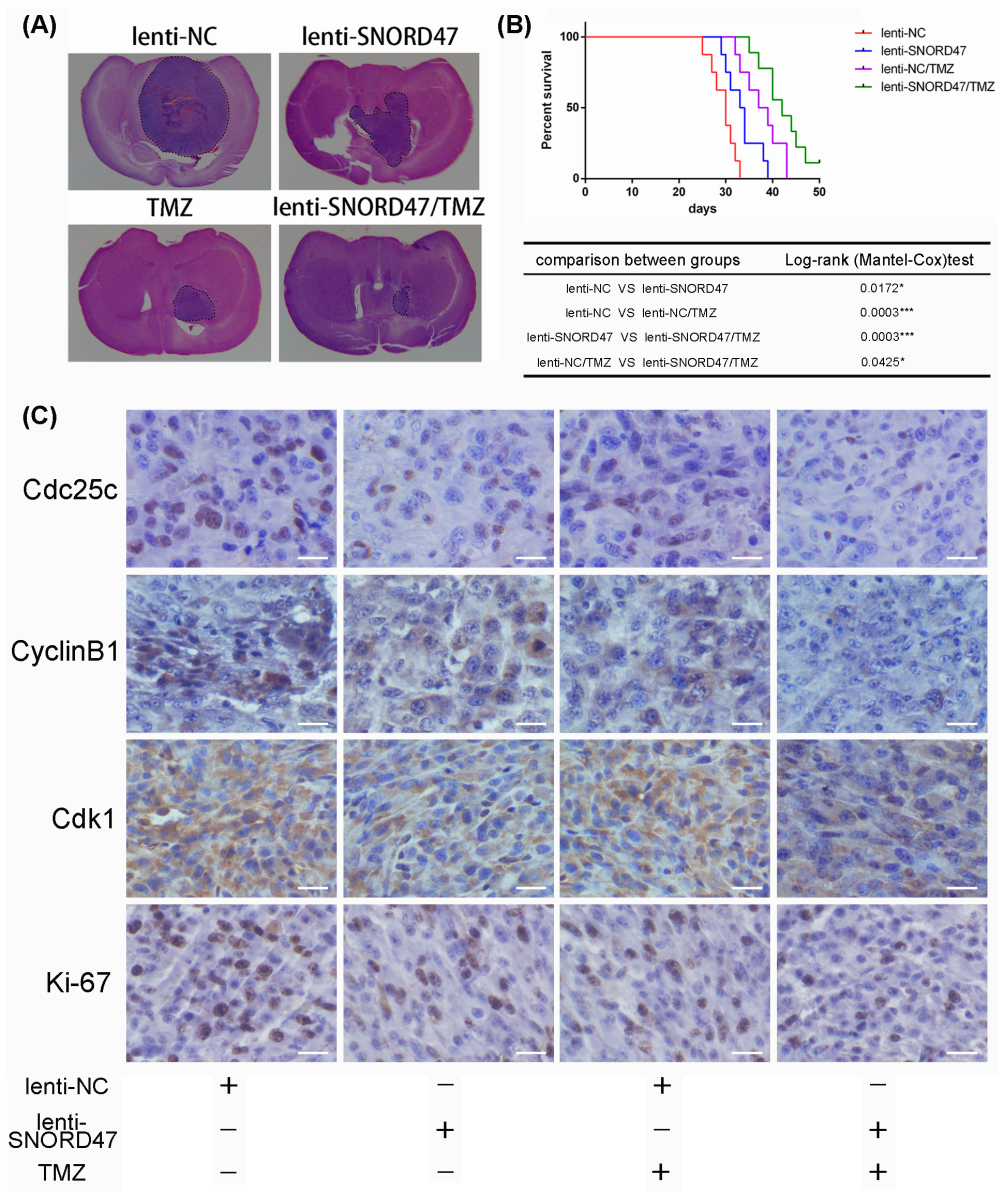
Overexpression of SNORD47 inhibited U87-MG orthotopic glioblastoma model and sensitized the temozolomide treatment *in vivo* **(A)** Representative images of HE staining of tissue from mice with orthotopic tumors derived from Lenti-NC- or Lenti-SNORD47 infected U87-MG cells with or without temozolomide treatment. **(B)** The survival of mice in NC and SNORD47 overexpression group with or without temozolomide treatment were demonstrated by Kaplan-Meier survival curves. Animal survival analysis (n=8, *P < 0.05, ***P<0.001, Log-rank (Mantel-Cox) test). **(C)** The immunohistochemical staining (IHC) analysis displayed the expression of the cell cycle-associated proteins in the indicated groups. Bar =20μm.

Next, to further determine whether SNORD47 had an antitumor effect and whether SNORD47 could strengthen the temozolomide-induced suppression of tumorigenesis *in vivo*, we constructed an intracranial nude mouse model in which mice were implanted with cells with or without SNORD47 overexpression lentivirus prior to treatment. As indicated, the results of hematoxylin and eosin staining of the brain tissues resected from the SNORD47-treated mice and the temozolomide-treated mice showed a significantly reduced tumor volume, and a longer survival time was also observed in these mice compared to the DMSO-treated mice (Figure 6A, 6B). The tumor size in mice in the SNORD47 group was slightly larger than in mice in the temozolomide group, and the survival time was slightly shorter. However, mice treated with a combination of SNORD47-temozolomide demonstrated a marked decrease in tumor size and a longer survival rate in comparison with mice treated with either SNORD47 or temozolomide alone, and especially when compared to the control mice (Figure 6A, 6B). Furthermore, IHC results indicated significantly downregulated expression levels of CDC25C, CyclinB1, and CDK1 in the SNORD47 and temozolomide groups and even more significantly decreased expression in the SNORD47-temozolomide group compared to the DMSO group (Figure 6C). There was no significant expression difference between the SNORD47 group and temozolomide group. Conversely, the expression level of Ki-67 was higher in the SNORD47 and temozolomide groups than in the SNORD47-temozolomide group, and especially higher in the control group (Figure 6C). In brief, these results revealed that SNORD47 represses tumorigenicity and strengthens the antitumor effect of temozolomide in glioma *in vivo*.

## DISCUSSION

SnoRNAs have been reported to play a stimulative or suppressive function in various cancer [2–4, 6, 7, 9, 11–21]. However, there have been few investigations associated with snoRNAs in glioma. It is known that SNORD47, a C/D box snoRNA, mainly modifies the methylation status of other RNAs. However, no reports have yet demonstrated the function of SNORD47 in cancer, including glioma. In the present study, we demonstrated the attenuated expression of SNORD47 in glioma clinical specimens. The patients with higher expression of SNORD47 obtained longer survival time compared with those with lower expression of SNORD47. The expression of SNORD47 in Grade IV glioma specimens was significantly inverse with HOTAIR, which is a negative prognostic factor in glioblastoma [10]. In orthotopic mouse models, SNORD47 significantly inhibited the growth of tumors and prolonged the survival time of mice. Taken together, these data indicated that SNORD47 plays a key suppressive role in gliomagenesis. In addition, this is the first demonstration that SNORD47 has the potential to inhibit the progression and development of glioma.

In this study, we found that SNORD47 significantly suppressed the growth and proliferation of glioma cells *in vivo* and *vitro*. Overexpression of SNORD47 led to downregulated expression of β-catenin and p-β-catenin. β-Catenin is a central component of the Wnt signaling pathway and can promote the proliferation of cancer cells [22]. Thus, we deduced that the possible explanation for the suppression of growth and proliferation induced by SNORD47 might be the inhibition of the β-catenin signaling pathway.

What's more, the flow cytometry analysis showed a G2-phase arrest after overexpression of SNORD47. Immunofluorescence staining of phospho-Histone H3, which is a marker of mitosis, demonstrated fewer cells undergoing mitosis in the lenti-SNORD47-treated group in comparison with the control group. The results of Western blot analysis and IHC analysis showed a lower expression of G2-phase specific proteins, including CyclinB1, CDK1, and CDC25C, after the lenti-SNORD47 treatment. It has been shown that CDC25C combining with the CyclinB1-CDK1 complex drive the cell cycle into mitosis from G2 phase [23, 24]. Therefore, our results indicated that lenti-SNORD47 impeded the entry of glioma cells into M phase, subsequently inhibiting the growth and proliferation of glioma cells to some extent.

The ectopic occurrence of EMT could induce tumorigenesis and promote tumor progression [25–29]. In this study, we found that SNORD47 repressed the EMT. The immunofluorescence results indicated that the cells’ cytoskeleton changed from stress fiber pattern to cortical pattern after treated with lenti-SNORD47. As a hallmark of mesenchymal phenotype, stress fiber could enhance the abilities of motion and migration of cancer cells, which results in the intensive invasion of cancer cells [29–31]. The results of EMT-related protein in Western bolt and immunofluorescence revealed the similar change of cytoskeleton. In addition, the total and nuclear level of β-catenin and p-β-catenin was also found to be lower after the lenti-SNORD47 treatment. These results suggested that SNORD47 impeded EMT-related gene transcription via suppressing the nuclear translocation and activation of β-catenin.

It is well known that temozolomide is characterized as a first-line chemotherapy. Temozolomide alone or combined with radiotherapy is given as the standard treatment for postoperative glioblastoma patients [32, 33]. As a DNA-alkylating agent, temozolomide plays a suppressive role in tumor by inducing DNA double-strand breaks and cell cycle arrest [34]. Dose-dependent hematologic toxicity and the resistance to temozolomide in cancer cells were two primary reasons limiting the usage of temozolomide in clinical therapies [35]. Therefore, we studied whether SNORD47 could ameliorate these deficiencies. We demonstrated that there was a synergistic effect between SNORD47 and temozolomide in inhibiting tumor growth *in vivo* and *vitro*. These results illustrated that SNORD47 augmented the sensitivity of glioma cells to temozolomide.

In summary, we demonstrated that SNORD47 inhibits glioma cell growth, proliferation, colony formation, invasion, and migration, induces G2-phase arrest *in vivo* and *vitro*. In addition, SNORD47 displayed a synergistic effect when combined with temozolomide and strengthened the sensitivity to temozolomide in glioma. This study offers new insights into the molecular mechanisms of glioma and provides a novel therapeutic strategy for glioma.

## MATERIALS AND METHODS

### Glioblastoma cell lines and cell culture

Human U87-MG and U251 glioblastoma cells were purchased from the China Academia Sinica Cell Repository (Shanghai, China). The glioblastoma cells lines were maintained in Dulbecco's modified Eagles medium (DMEM; Corning) supplemented with 10% fetal bovine serum (FBS; Gibco), and incubated at 37°C in 5% CO_2_ in a humidified chamber.

### Reagents

temozolomide was purchased from Sigma-Aldrich Co (St. Louis, MO, USA). Antibodies against CDK4, CyclinD1, Ki-67 and E2F1 were purchased from Santa Cruz Biotechnology (Santa Cruz, CA, USA). Antibodies against p27, pRb, E-Cadherin, N-Cadherin, β-Catenin, p-β-Catenin, Twist, Snail, Vimentin, and Slug were purchased from Cell Signaling Technology (CST, USA). Antibodies against F-actin and β-Tubulin and the fluorescent anti-rabbit or anti-mouse IgG secondary antibodies were purchased from Thermo Fisher Scientific (USA). Antibodies against Cdk1, CyclinB1, GAPDH, MMP2, and MMP9 were purchased from Proteintech Group (USA). Anti-phosphorylated Histone H3 and Cdc25c was purchased from Abcam (USA). 4,6-Diamino-2-phenylindole (DAPI) was purchased from Solarbio company (Beijing, China). The Nuclear and Cytoplasmic Protein Extraction Kit was purchased from Beyotime (China). 5-ethynyl-20-deoxyuridine (EdU) Kit was purchased from Ribobio company (Guangzhou, China).

### Clinical specimens

The fresh resected tissue was immediately snap-frozen in liquid nitrogen. The total RNA was subsequently extracted. Glioma specimens were collected from 124 patients who underwent glioma resection at the Second Affiliated Hospital of Nanchang University between January 2002 and December 2009. Informed consent was obtained from each patient, and the study protocol was approved by the Ethics Committee of the Second Affiliated Hospital of Nanchang University.

### Lentiviral infection and RNA interference

Lentiviral vectors expressing nonsense control (NC), SNORD47 (NCBI Reference Sequence: NR_002746.1) were generated by GenePharma (Shanghai, China). Cell infections were carried out according to GenePharma's recommendations.

### RNA extraction and quantitative real-time PCR

Total RNA was extracted using TRIzol (Invitrogen) and the RNA concentration and quality were measured using a NanoDrop ND-1000 spectrophotometer (NanoDrop Technologies). qRT-PCR assays were performed to measure the expression levels of SNORD47, and GAS5 according to the manufacturer's instructions. Real-time PCR was conducted using the SYBR Green PCR Master Mix (Applied Biosystems) according to the manufacturer's instructions. Primers specific for each of the signaling molecules were designed using NCBI/Primer-BLAST and used to generate the PCR products. The expression level of RNU6 was used to quantify the expression of SNORD47, while expression levels of GAPDH were used to normalize and quantify the expression level of HOTAIR. For the quantification of gene amplification, quantitative real-time PCR was performed by using the DNA Engine Opticon 2 Two-Color Real Time PCR detection system (Bio-Rad Laboratories) in the presence of SYBR-Green. Quantitative real-time PCR data were analyzed by the comparative Ct method, shown as fold change (2^-ΔΔCt^)[36]. The following gene-specific primers were used: SNORD47 (F: 5’-CCAATGATGTAATGATTCTGCCA-3’; R: 5’-ATACCAACAAGTGCTGAGGAAGTG-3’); GAS5 (F: 5’-CTTCTGGGCTCAAGTGATCCT-3’; R: 5’-TTGTG CCATGAGACTCCATCAG-3’); HOTAIR (F: 5’-GAGAAA AGGCTGAAATGGAGGACC-3’; R: 5’-TCTTCCCTCC TCTGGCTCTCTCTC-3’); GAPDH (F: 5’-CTCAAGGG CATCCTGGGCTAC-3’; R: 5’-CAGCCCCAGCGTCAAA GGT-3’); RNU6 (F: 5’-ATTGGAACGATACAGAGAA GATT-3’; R: 5’-GGAACGCTTCACGAATTTG-3’).

### Cell cycle analysis

U87-MG and U251 cells were infected with lenti-SNORD47 or lenti-nonsense control with or without subsequent temozolomide treatment. After fixation in 70% ethanol and RNase A treatment, cells were stained with propidium iodide. DNA content was analyzed by flow cytometry. In addition, the percentages of cells within each phase of the cell cycle were analyzed using FlowJo software.

### Cell viability assay

The cell viability was evaluated by CCK-8 assay. 2*10^3^ cells were plated in 96-well plates and treated as indicated. After the treatment, one hundred and ten microliters of culture medium containing ten microliters of CCK-8 solution was added to each well and incubated for an additional 2 h at 37°C. The optical density (OD) of each well at 450 nm was recorded on a microplate reader. The cell viability (% of control) is expressed as the percentage of (ODtest − ODblank)/(ODcontrol − ODblank).

### Wound-healing assay

The cells were grown to 80% to 90% confluency in 6-well plates. Artificial wounds were generated by scraping a pipette tip across the cell surface. The cell movement during wound closure was measured by phase contrast photography at 37°C for incubations of 0 and 24 h, and ten randomly selected wound areas were analyzed.

### Transwell assay

The invasion assays were performed using Matrigel-coated invasion plates to measure the invasive capacity of glioblastoma cells. The cells were seeded in the upper chambers containing serum-free DMEM in a 24-well plate, and DMEM with 5% FBS was placed in the lower chambers. After a 24 h incubation, the cells were removed from the upper surfaces of the invasion membranes, and the cells on the lower surface were stained with crystal violet. The average number of cells per field was determined by counting the cells in 3 random fields per well. Images from each well were captured by microscopic analysis using a camera-equipped Olympus Vanox microscope.

### Colony formation assay

Cells were collected and counted, and then seeded in 6-well plates at a density of 500 cells per well in triplicate for each cell line. The culture medium was replaced every 3 days after seeding. Colonies were counted only if they contained more than 50 cells, and the number of colonies was counted approximately 15 days after seeding. The cells were stained using crystal violet.

### Western blot

Total protein and nuclear proteins of the glioma cells were isolated according to the manufacturer's instructions. The protein samples were resolved via sodium dodecyl sulfate-polyacrylamide gel electrophoresis (SDS-PAGE) and transferred onto PVDF membranes via electroblotting. The membranes were then incubated with primary antibodies against CDK4, CyclinD1, E2F1, pRb, p27, Cdk1, Cdc25c, CyclinB1, GAPDH, MMP2, MMP9, E-Cadherin, N-Cadherin, β-Catenin, p-β-Catenin, Twist, Snail, Vimentin, Slug, and pH3 overnight at 4°C. The membranes were then incubated with the appropriate secondary antibodies. Immunoreactive proteins were detected using an ECL Western Blotting Substrate.

### Immunofluorescence analysis

Cultured U87-MG and U251 cells were fixed with 4% paraformaldehyde (Solarbio, Beijing) for 20 min at room temperature. The cells were then treated with 0.1% Triton X-100 in PBS for 20 min. Non-specific binding was blocked by incubating cells with 5% BSA in PBS for 30 min at 37°C. The slides were then incubated with primary antibodies against F-actin, anti-β-Tubulin, anti-pH3, anti-E-Cadherin, and anti-N-Cadherin at a dilution of 1:100 overnight at 4°C. After the slides were washed, they were incubated with the fluorescent secondary antibody (dilution 1:500) at room temperature for 1 h. The slides were then incubated with DAPI. Microscopy analysis was performed using a fluorescence microscope.

### EdU assay

The proliferation of cells was measured by EdU assay according to the manufacturer's instruction. First, the cells were incubated with 5-ethynyl-20-deoxyuridine (EdU) at 37°C in 5% CO2 in a humidified chamber for 2 hours. After washed with PBS, the cells were treated with 1×Apollo® reaction reagent at room temperature for 30 min. Then, the DNA contents of the cells in each well were stained with Hoechst 33342 for 30 min at room temperature and visualized using a fluorescence microscope.

### Nude mouse tumor xenograft model and treatment

The mice were randomly divided into four groups. Four-week-old female nude mice (Cancer Institute of the Chinese Academy of Medical Science) were stereotactically implanted using cranial guide screws with 5*10^5^ U87-MG cells transduced with lenti-NC and lenti-SNORD47 to create intracranial gliomas. One week after the implantation, each group was intraperitoneally injected with DMSO or temozolomide (dissolved in DMSO, 20 mg/kg) every two days. The study followed the internationally recognized guidelines on animal welfare and local and national regulations. The overall survival curves were plotted according to the Kaplan-Meier method.

### Hematoxylin-eosin (H&E) staining and immunohistochemistry analysis

The paraffin-embedded tissue sections were stained with hematoxylin-eosin (H&E) and then analyzed for examination of Cdc25c, Cdk1, CyclinB1 and Ki-67 expression by IHC as previously described [37]. Briefly, for immunohistochemistry analysis, sections were incubated with primary antibodies (1:100 dilutions) overnight at 4°C and then treated with a biotin-labeled secondary antibody (1:100 dilutions) for 1 h at room temperature. After the sections were washed with PBS, they were incubated with 3, 3’-diaminobenzidine (DAB), counterstained with hematoxylin, and visualized using a microscope.

### Statistical analysis

Statistical software (SPSS) version 20.0 was used for the analysis of statistical significance. All data were represented as the mean±SD. The t-test was used to determine differences between 2 groups. One-way ANOVA was used to test for differences among at least 3 groups, and a least significant difference post-hoc test was used to obtain individual P values followed by ANOVA. Kaplan-Meier analysis and log-rank tests were used to evaluate the difference in survival. A *P-*value <0.05 was considered significant.

## Abbreviations

snoRNAs: small nucleolar RNAs
EMT: epithelial-mesenchymal transition
GAS5: growth arrest-specific transcript 5
